# Extracellular Superoxide Dismutase in Acute Respiratory Distress Syndrome: Pathogenic Mechanisms and Therapeutic Implications

**DOI:** 10.3390/antiox15020249

**Published:** 2026-02-13

**Authors:** William Osier, Eva S. Nozik, Christina Sul

**Affiliations:** 1 University of Colorado Anschutz School of Medicine, Aurora, CO 80045, USA; william.osier@cuanschutz.edu; 2 Cardiovascular Pulmonary Research Group, Department of Pediatrics and Medicine, University of Colorado Anschutz Medical Campus, Aurora, CO 80045, USA; 3 Department of Pediatrics, Section of Critical Care, University of Colorado Anschutz Medical Campus, Aurora, CO 80045, USA

**Keywords:** EC-SOD, acute lung injury, ARDS

## Abstract

The lung is highly susceptible to oxidative stress because of its exposure to high oxygen tension and environmental stressors, making tight regulation of the redox environment essential for homeostasis and disease pathogenesis. Extracellular superoxide dismutase (EC-SOD, *sod3*) is an important antioxidant enzyme in the lung that catalyzes the dismutation of superoxide into hydrogen peroxide and oxygen, thereby regulating the redox environment of the extracellular matrix, cell surfaces, and lining fluids of the lung. This review summarizes the structural features, post-translational regulation, genetic variations, and cellular sources of EC-SOD, with a particular focus on its role in acute respiratory distress syndrome (ARDS). We highlight evidence demonstrating that loss of EC-SOD exacerbates dysregulated immune responses, whereas enhanced EC-SOD activity confers protection in multiple experimental models of acute lung injury. We also discuss how inflammatory signaling, epigenetic regulation, aging, and genetic polymorphisms in the *sod3* gene influence EC-SOD expression and function. Finally, we review emerging therapeutic strategies, including SOD mimetics and mRNA-based approaches, and address the challenges associated with non-specific antioxidant therapies in ARDS. Collectively, the data position EC-SOD as a central regulator of extracellular redox signaling and a promising, mechanism-driven therapeutic target in acute lung injury and ARDS.

## 1. Structural and Functional Characteristics of EC-SOD

The lung is uniquely vulnerable to oxidative stress due to its continuous exposure to high oxygen tension and environmental stressors, making redox balance a critical determinant of pulmonary health and disease. Redox reactions are essential for aerobic life and are controlled within specific cellular compartments by redox enzymes. Notable redox enzymes include catalase, glutathione peroxidase, glutathione reductase, glutaredoxins, peroxiredoxins, thioredoxins, and the focus of this review, superoxide dismutases (SOD). SOD enzymes catalyze the dismutation of superoxide (O_2_^•−^) into hydrogen peroxide (H_2_O_2_) and molecular oxygen (O_2_). Three isoforms of SOD have been identified in mammals: copper zinc SOD (CuZn-SOD, *sod1*), manganese SOD (Mn-SOD, *sod2*), and extracellular SOD (EC-SOD, *sod3*) [1,2,3]. CuZn-SOD is present in the cytoplasm, nucleus, and peroxisomes of all mammalian cells [4,5], whereas Mn-SOD is found primarily in mitochondria [6,7,8]. In contrast to the other isoforms, a secretory signal peptide (1–18 aa) targets EC-SOD for secretion [9,10,11], where it modulates the extracellular redox environment, including the extracellular matrix, cell surfaces, and lining fluids [3,12,13].

**Figure 1:**
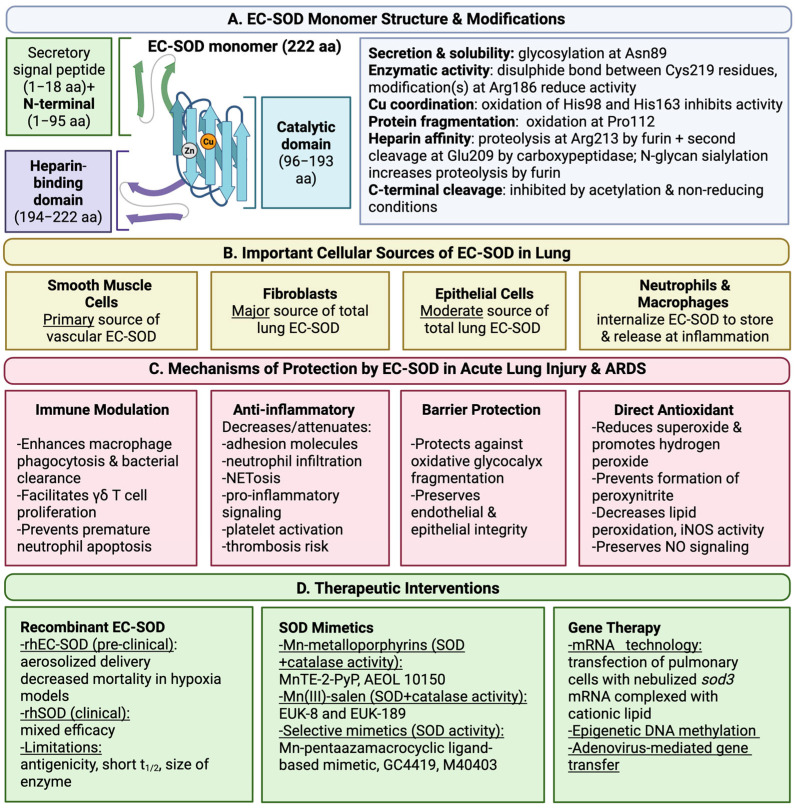
**Summary of EC-SOD monomer domains, post-translational modifications, cellular sources in the lung, protective mechanisms in the pathogenesis of acute lung injury and ARDS, and therapeutic interventions.** Abbreviations: aa—amino acids; Asn—asparagine; Cys—cysteine; Arg—arginine; His—histidine; Pro—proline; Glu—glutamate; NET—neutrophil extracellular trap; iNOS—inducible nitric oxide synthase; NO—nitric oxide; rh—recombinant human; t_1/2_—half-life.

EC-SOD is a hydrophobic glycoprotein that most commonly exists as a tetramer (approximately 135 kDa), composed of four functional subunits (approximately 30 kDa each) [3,12,13,14]. Dimers [14,15] and larger multimers [16,17] have also been reported. Each subunit contains one copper atom and one zinc atom, which are required for enzymatic activity [12,18]. Tetrameric EC-SOD forms through noncovalent interactions between two EC-SOD dimers [16]. Each dimer contains two EC-SOD monomers that are linked by a disulfide bond between C-terminal cysteine residues (Cys219) [16]. EC-SOD monomers adopt active (aEC-SOD) or inactive (iEC-SOD) conformations, dictated by intrapeptide disulfide bridging [19]. The primary amino acid sequence of EC-SOD is composed of 240 aa, with the first 18 aa encoding a secretory signal peptide [9]. Removal of the signal peptide yields mature EC-SOD, which is composed of the remaining 222 aa [9]. Mature EC-SOD contains three functional domains: an N-terminal domain (1–95 aa), a catalytic domain (96–193 aa), and an extracellular matrix-binding region (194–222 aa) [9,20] (Figure 1).

The catalytic domain of EC-SOD is homologous to the cytoplasmic isoform CuZn-SOD, sharing approximately 50% sequence identity [9]. EC-SOD activity is copper-dependent, and copper delivery is facilitated by the chaperone Atox-1 [21]. H_2_O_2_ can inhibit EC-SOD activity by oxidizing copper-coordinating histidine residues (His98 and His163) [22]. Interestingly, H_2_O_2_ oxidizes homologous residues in CuZn-SOD, leading to a similar loss of enzymatic function [22,23,24]. Likewise, Arg186 in EC-SOD is homologous to the completely conserved CuZn-SOD residue Arg143 [9,25], and arginine modification impairs the activity of both enzymes [25,26]. It is also important to note that oxidation of the catalytic residue Pro112 can induce EC-SOD fragmentation [22] (Figure 1).

The N-terminal domain contains the only N-glycosylation site in EC-SOD (Asn89) [9]. The structure of the N-glycan can vary, but the predominant form is a disialylated, complex, core-fucosylated biantennary glycan [27,28,29]. N-glycosylation is critical for maintaining solubility [10] and enabling EC-SOD secretion [11]. Of note, the N-terminal residue Val28 is critical for EC-SOD tetramerization [30] (Figure 1).

The extracellular matrix-binding region contains a C-terminal heparin-binding domain, which is not present in other SOD isoforms [9,31,32]. The heparin-binding domain is composed of polycationic residues (210–216 aa) [31,32] that form electrostatic interactions with heparin [33,34], as well as extracellular matrix components, including heparan sulfate [34], type I collagen [20], and hyaluronan [35]. Three forms of EC-SOD, which are classified based on heparin affinity as type C (high affinity), type B (intermediate affinity), and type A (no affinity), are present in human plasma at nearly equal concentrations [33], whereas type C is the primary form present in the extracellular matrix [36,37].

Differences in heparin affinity result from intracellular removal of the heparin-binding domain via proteolysis at Arg213 (which is involved in the R213G variant discussed below) by the proteolytic enzyme, furin, followed by a second cleavage event at Glu209 involving an unknown carboxypeptidase [14,31,38,39]. Type C and type A are homodimers distinguished by complete retention (type C) or absence (type A) of the heparin-binding domain [31]. In contrast, type B EC-SOD exists as a homodimer or heterodimer, depending on the configuration and structure of each subunit [31]. Heparin-binding domain cleavage is inhibited under non-reducing conditions [39] and following acetylation by S-acetyl glutathione or acetyl coenzyme A [40]. Acetylation does not alter enzymatic function, and deacetylation by SIRT1 or SIRT3 fully restores furin cleavage [40]. N-glycan sialylation has also been shown to increase furin-mediated cleavage of the heparin-binding domain [28]. Overall, these findings suggest that C-terminal proteolysis of EC-SOD is an organized process regulated by the intracellular redox environment and post-translational modifications.

Furthermore, the heparin-binding domain facilitates clathrin-dependent endocytosis of EC-SOD by binding to low-density lipoprotein receptor-related protein (LRP) and cell-surface heparan sulfate [41,42,43,44]. There is also evidence that the heparin-binding domain functions as a redox-regulated nuclear localization signal, capable of directing both endogenous and exogenous EC-SOD to the nucleus [41,42]. While these results have been challenged on the basis of experimental methodology [43], nuclear localization of EC-SOD has been observed in other models [45,46], with one study demonstrating a significant reduction in markers of oxidative DNA damage [45]. Interestingly, all three types of EC-SOD are susceptible to non-enzymatic glycosylation in vivo, with type B and type A displaying the highest degrees of glycosylation [47]. Given that the heparin-binding domain is removed prior to secretion [14,31,38,39], these findings suggest that EC-SOD is susceptible to extracellular glycosylation, consistent with the aforementioned studies reporting nuclear localization of post-translationally modified EC-SOD [41,42]. Collectively, these observations suggest that EC-SOD may have intranuclear functions, warranting further research.

EC-SOD has also been localized to the cytoplasm in a variety of cell types [13,41,42,45,46,48,49,50,51,52,53,54,55,56,57], accounting for approximately 5% of total cellular SOD activity on average [13,48]. Despite relatively low activity, intracellular EC-SOD significantly reduces cellular reactive oxygen species (ROS), thereby attenuating myocardial reperfusion injury [46,53], estrogen-induced breast cancer progression [45], and telomere shortening [51].

## 2. Tissue Localization and Cellular Sources of EC-SOD

EC-SOD is the major isozyme of SOD in human arteries and is also highly expressed and active in the lung [13,58,59]. This is relevant because the lungs are directly exposed to atmospheric oxygen, and disruption of EC-SOD has long been predicted to contribute to oxygen toxicity, particularly in patients with ARDS requiring supplemental oxygen [60].

**Figure 2:**
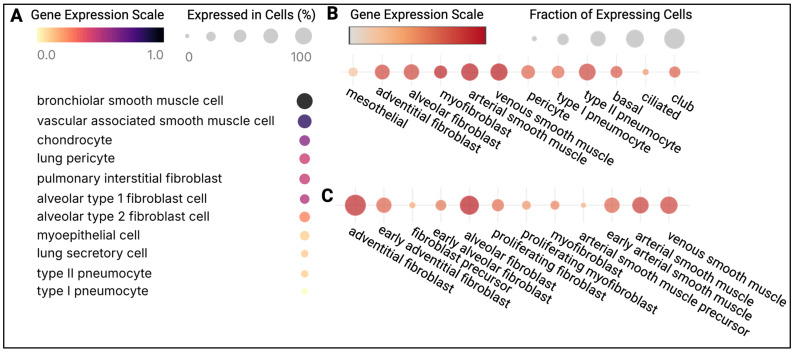
**High expression of the EC-SOD gene *sod3* in lung smooth muscle, fibroblast, and epithelial cell populations.** Smooth muscle cells are the highest expressors of *sod3* in the healthy (**A**) adult and (**B**) neonatal human lung. Fibroblasts and smooth muscle cells are the highest expressors of *sod3* in the (**C**) neonatal murine lung. Sources: (**A**) the Integrated Human Lung Cell Atlas (HLCA) v1.0, www.humancellatlas.org, URL accessed on 12 December 2025, and (**B**,**C**) the Neonatal Single Cell Compressed Atlas of the Lung, www.neonatallungatlas.org, URL accessed on 12 December 2025.

In the human lung, EC-SOD is concentrated mainly in the extracellular matrix and on the cell surface of large vessels and airways, with lower levels near alveoli, capillaries, and bronchiolar epithelial junctions [37]. Gene expression data from human lung tissue is searchable in the Human Lung Cell Atlas, the first integrated single-cell atlas of the lung [61], and shows that adult *sod3* expression is highest in the cell types listed in Figure 2A, most notably smooth muscle cells, fibroblasts, and epithelial cells. These findings are consistent with the Neonatal Human Lung Atlas [62,63], which identifies arterial and venous smooth muscle cells and myofibroblasts as major expressors of *sod3*, and to a lesser extent, adventitial and alveolar fibroblasts and epithelial cells in neonates (Figure 2B). In mice, vascular smooth muscle cells and fibroblasts are the two major cellular sources of lung *sod3*, with moderate expression by airway epithelial cells (Figure 2C). Importantly, depletion of *sod3* in smooth muscle cells significantly reduces vascular EC-SOD content without altering total lung EC-SOD levels, indicating the significant contributions of lung fibroblasts and epithelial cells to maintaining basal lung EC-SOD production [64,65,66]. Endothelial cell *sod3* expression is insufficient in both species to significantly contribute to pulmonary EC-SOD levels [61,62,63,67,68,69,70]. The low production of EC-SOD by endothelial cells has been attributed to epigenetic modifications of *sod3* at multiple locations, including the sole binding site for the transcription factors Sp1 and Sp3, which are critical for initiating transcription [69,70,71,72,73,74]. Similar epigenetic repression of *sod3* has been observed in pulmonary artery smooth muscle cells from patients with idiopathic pulmonary arterial hypertension [75]. Of note, inhibiting or reversing epigenetic modifications to *sod3* induces significant EC-SOD production in endothelial cells, possibly via the JAK2/STAT3 and p44/p42 ERK1/2 pathways [69,70,71]. Given the clinical relevance of *JAK2* mutations, further research is warranted.

Furthermore, IFN-γ and IL-6 transcriptionally upregulate *sod3* in smooth muscle cells and fibroblasts [69,76]. Interestingly, both cytokines induce endothelial O_2_^•−^ production [77], and IFN-γ positively regulates gp91phox synthesis in smooth muscle cells, resulting in additional O_2_^•−^ production via NADPH oxidase 2 [69,78,79]. NADPH oxidase 2 is a transmembrane enzyme that transfers electrons from cytosolic NADPH to molecular oxygen, generating O_2_^•−^. Multiple vasoactive molecules also upregulate *sod3* in smooth muscle cells, including angiotensin II [80], which additionally activates vascular NADPH oxidase 2 and increases de novo synthesis of NADPH oxidase 2 subunits in smooth muscle cells [79]. Nitric oxide (NO^•−^), a critical molecule for regulating arterial tone, upregulates EC-SOD production via the cGMP/protein kinase G pathway in vascular smooth muscle cells [81]. Importantly, vascular smooth muscle cells comprise distinct subpopulations [82] and exhibit phenotypic plasticity in response to the extracellular environment, leading to differential *sod3* expression [80]. TNF-α and TGF-β negatively regulate *sod3* in smooth muscle cells and fibroblasts [69,76,83].

## 3. Acute Respiratory Distress Syndrome

Acute respiratory distress syndrome (ARDS) is a life-threatening condition affecting adults and children. The Berlin Definition outlines the diagnostic criteria for adult ARDS and classifies disease severity as mild, moderate, or severe based on the degree of hypoxemia [84]. ARDS develops in the context of a known clinical injury, most commonly pneumonia, aspiration, or sepsis, which together account for more than 85% of ARDS cases [85,86]. Mortality rates differ between mild (27–31.4%), moderate (32–34.9%), and severe (45–46.1%) ARDS [85,86]. Despite the long-standing recognition of ARDS and its high mortality, proven therapeutic strategies are limited to lung-protective ventilation strategies and judicious fluid management.

Of note, limitations of the Berlin Definition in children prompted the development of the PALICC definition, a distinct set of guidelines outlining the diagnosis and management of pediatric acute respiratory distress syndrome (PARDS), in 2015 [87]. Subsequent studies identified variability in PALICC implementation across pediatric intensive care units (PICUs) [88,89,90], and nonadherence to PALICC recommendations was associated with increased mortality in patients with PARDS [90,91,92]. The 2015 PALICC definition also demonstrated limitations in resource-limited settings [90]. Collectively, these findings informed the 2023 revision, culminating in the current PALICC-2 definition and guidelines [90].

The pathophysiology of ARDS is complex. Broadly, isolated or combined injury to the alveolar epithelium or pulmonary endothelium triggers a hyperinflammatory response that disrupts the alveolar–capillary barrier, resulting in proteinaceous fluid accumulation within the alveoli. Clinically, this process manifests as pulmonary edema and impaired gas exchange, resulting in often severe hypoxemia. Innate immune cells are central to ARDS pathogenesis. Multiple cell types, including alveolar macrophages, express pattern-recognition receptors [93]. Activation of surface pattern-recognition receptors on alveolar macrophages by damage-associated molecular patterns released during cell injury or pathogen-associated molecular patterns present on microbes induces cytokine (e.g., TNF-α, IL-6, IL-17) and chemokine (e.g., IL-8, CCL2, and CCL7) release [85]. These proinflammatory molecules recruit neutrophils and monocytes from the systemic circulation to the pulmonary vasculature and facilitate extravasation into the airways by upregulating endothelial adhesion molecule expression and increasing vascular permeability [94,95,96,97,98]. Importantly, O_2_^•−^ facilitates inflammatory molecule production [99,100,101,102] and downstream cellular responses [103,104,105,106,107,108,109].

Recruited neutrophils cause further injury to the alveolar–capillary barrier and alveolar epithelium via marked ROS production, including NADPH oxidase 2-derived O_2_^•−^ [110,111]. While O_2_^•−^ is a poor oxidant itself, it has strong reducing capacity and generates several potent oxidizing agents. Protonation of O_2_^•−^ forms the perhydroxyl radical (HOO^•^), a key initiator of lipid peroxidation [112]. Nitric oxide (NO^•^) present within the vasculature reacts rapidly with O_2_^•−^ to produce peroxynitrite (HNOO^−^), a strong oxidizing agent that can generate secondary radicals, including nitrogen dioxide (^•^NO_2_) and the hydroxyl radical (OH^•^), all of which oxidize lipids, proteins, and DNA [113]. Depletion of endogenous NO^•^ also results in vasoconstriction, decreasing pulmonary blood flow, thereby worsening hypoxemia. Additionally, O_2_^•−^ facilitates OH^•^ production by reducing ferric iron (Fe^3+^) to its ferrous (Fe^2+^) state, which is subsequently oxidized by H_2_O_2_ to form OH^•^ and hydroxyl anion (OH^−^) via Fenton chemistry [114,115,116,117]. OH^•^ depolymerizes components of the endothelial glycocalyx, releasing additional chemoattractants, including type I collagen [20], heparan sulfate [118], hyaluronan [35], and syndecan-1 [119]. Importantly, loss of alveolar epithelial heparan sulfate is sufficient to induce alveolar–capillary barrier dysfunction [98], and glycocalyx degradation increases vascular permeability [120,121,122]. Likewise, O_2_^•−^ positively regulates endothelial TNF-α production [103], and TNF-α increases O_2_^•−^ production via NADPH oxidase, ultimately decreasing endothelial cell–cell interactions by facilitating internalization of vascular endothelial cadherin [105,106]. O_2_^•−^ also induces endothelial apoptosis by activating caspase-3 [109,123], and TNF-α activates heparanase [97].

Similar findings have been observed in translational ARDS studies [97,124,125,126,127]. For example, plasma heparanase activity was significantly higher in mechanically ventilated patients relative to healthy controls, as well as in cases of nonpulmonary sepsis compared with other etiologies of acute respiratory failure [97]. Glycosaminoglycans have been used to phenotype ARDS patients in other studies as well [124,125,126,127]. Of note, acute kidney injury significantly increases ARDS mortality [128,129,130] and is mediated by NADPH oxidase-derived O_2_^•−^ production and heparan sulfate degradation [131,132,133,134,135]. Measuring urinary glycosaminoglycans and heparanase activity demonstrates clinical utility in predicting renal dysfunction and outcomes in septic shock and ARDS [136].

Activated neutrophils also damage the alveolar–capillary barrier by forming neutrophil extracellular traps (NETs), a process characterized by potent NADPH oxidase 2 activity and marked generation of ROS. NETs are extracellular fibers composed of decondensed chromatin with granule and nuclear constituents, including proteases, DNA, and histones, that assist with pathogen clearance and virulence factor degradation [137,138]. Classical, or suicidal, NET formation, also referred to as NETosis, is a lytic process that requires NADPH oxidase 2 and results in cell death [139,140]. In contrast, vital NET formation is non-lytic and independent of NADPH oxidase 2 [141,142]. NET formation is induced by a variety of factors, including endotoxin (lipopolysaccharide) and IL-8 [137], in addition to ROS [139] and endotoxin-activated platelets [143]. Endotoxin-activated platelets also induce neutrophil degranulation directly through a TLR4-mediated process [143]. Importantly, NET formation is significantly increased in patients with ARDS and correlates with increased mortality and disease severity [144].

EC-SOD impacts the pathophysiology of ARDS at multiple levels by regulating the extracellular redox environment. EC-SOD has been shown to attenuate proinflammatory signaling and responses in multiple models of acute lung injury [145,146,147,148]. EC-SOD overexpression inhibits the upregulation of endothelial adhesion molecules and attenuates pulmonary inflammatory cell infiltration [145,147]. EC-SOD is protective against ROS-induced cytotoxicity in acute lung injury, as evidenced by significant reductions in lipid peroxidation [145,146,149,150], iNOS activity [146,151], and nitrotyrosine accumulation [83,146,151] (Figure 1).

Activated neutrophils and macrophages release EC-SOD at sites of inflammation, thereby attenuating local oxidative stress [152,153]. Additionally, EC-SOD quenches intracellular ROS generated by innate immune cells during the oxidative burst [54,57]. Of note, *sod3* is expressed in alveolar macrophages but not in neutrophils [55,67,152,153,154,155,156]. Nevertheless, both cell types can acquire EC-SOD through endocytosis and subsequently store the protein within secretory vesicles [54,55,56,152,153,156]. Activated inflammatory cells also display increased levels of surface-bound EC-SOD [54,55,56,156]. Overall, leukocytes are a major source of EC-SOD during acute inflammation [152,153,156], despite their minimal contribution to basal lung EC-SOD production [61,62,63,67,68,69,70] (Figure 1).

Furthermore, EC-SOD augments the innate immune response by enhancing phagocytosis in macrophages [54]. Interestingly, the heparin-binding domain is bacteriostatic under nonphysiological salt concentrations, with slightly higher activity against Gram-negative bacteria compared to Gram-positive strains [157], and treatment with nebulized EC-SOD mRNA complexed with lipofectamine significantly reduced bronchoalveolar *E. coli* growth in rats [158]. EC-SOD is also present in lipid rafts in LPS-activated macrophages [55,56]. Of note, lipid raft-associated EC-SOD promotes VEGF signaling in endothelial cells via oxidative inactivation of DEP-1/PTP1B [159,160]. Additionally, membrane-bound EC-SOD attenuates intracellular ROS levels, which has been shown to reduce tumor growth [52] and decrease heparanase expression [160]. Collectively, these findings suggest that the translocation of EC-SOD to lipid rafts in activated macrophages may have functional significance, such as signal transduction or pathogen recognition, and therefore warrants further research. EC-SOD also promotes IL-17A + γδ T cell proliferation by preventing premature neutrophil apoptosis [57]. IL-17A + γδ T cells are important sources of IL-17 during bacterial infections and recruit neutrophils to the lungs [161,162]. γδ T cells are part of the adaptive immune system, originate in the thymus, and demonstrate memory functions [161,162,163].

Redistribution of EC-SOD to the extracellular fluids, such as plasma and bronchoalveolar lining fluid, has been shown to reduce NETosis in *Staphylococcus aureus* (*S. aureus*)-induced acute lung injury [164]. In the same model of *S. aureus* pneumonia, increased circulating EC-SOD attenuated platelet activation, inhibited platelet-neutrophil aggregation, and prevented pulmonary accumulation of both cell types [165], all of which could contribute to the observed reduction in NET formation and acute lung injury. Given that NETs are prothrombotic [166], EC-SOD may also be protective against thrombosis. Importantly, increased pulmonary EC-SOD activity significantly reduces neutrophil influx [145,146,147,148,149], acute lung injury histologic scores [145,146,148,149], and mortality [83,145] in multiple other models of acute lung injury. Conversely, significant reductions in pulmonary EC-SOD have been reported following exposure to endotoxin [83,147,148,151] and hyperoxia [167] with corresponding increases in neutrophil influx [147,148], acute lung injury severity [148,151], and higher mortality [83,148]. EC-SOD also prevents oxidative fragmentation of the glycocalyx and thereby attenuates neutrophil chemotaxis [20,35,118,119]. Intriguingly, heparan sulfate functions as a mechanoreceptor on endothelial cells, regulating NO^•^ production in response to shear stress [168]. Theoretically, EC-SOD could reduce pulmonary vasoconstriction by preventing degradation of heparan sulfate, which could improve pulmonary blood flow and reduce right heart strain. Consistent with this hypothesis, EC-SOD maintains renal blood flow in sepsis models [169] and has been shown to downregulate heparanase in a breast cancer model [160]. ARDS can be complicated by acute pulmonary hypertension. Although the contribution of EC-SOD has not been examined in the development of this complication, it is worth noting that there is extensive data on the role of EC-SOD in the setting of chronic hypoxic pulmonary hypertension [170,171,172]. Collectively, it is highly likely that loss of EC-SOD from the pulmonary vasculature and lungs plays a significant role in ARDS progression.

**Figure 3:**
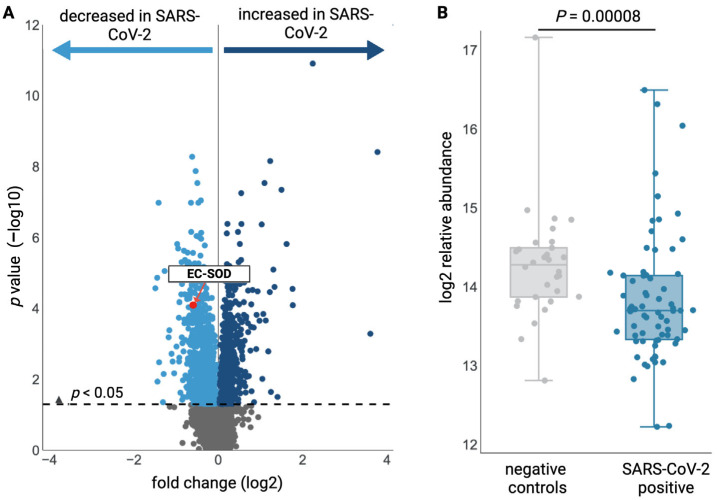
**EC-SOD levels are decreased in patients with SARS-CoV-2 infection.** Plasma samples from participants with confirmed SARS-CoV-2 infection (*n* = 69) and COVID-19-negative controls (*n* = 30) were analyzed using SOMAscan^®^ assays to generate plasma proteome profiles for the detection of epitopes corresponding to 3500+ unique proteins. (**A**) Volcano plot depicting changes in the plasma proteome in response to SARS-CoV-2 infection. (**B**) Plasma EC-SOD levels. Differences between groups were analyzed using the Kolmogorov–Smirnov test. Source: The COVIDome project, University of Colorado Anschutz Medical Campus, covidome.org, URL accessed on 17 December 2025 [173].

Translational EC-SOD studies are limited. Here, we present previously unpublished data demonstrating significantly lower plasma EC-SOD levels in non-critically ill hospitalized patients with SARS-CoV-2 infection compared to healthy controls (Figure 3) [173]. Similar findings of reduced plasma EC-SOD levels have been reported in pediatric patients hospitalized with *Mycoplasma pneumoniae* [174]. Intriguingly, SARS-CoV-2 and *M. pneumoniae* are both intracellular pathogens and elicit similar inflammatory responses, as evidenced by elevations in serum IL-8 and TNF-α [174,175]. TNF-α negatively regulates EC-SOD expression [69,76,83], offering one possible mechanism to explain these findings. However, this explanation is likely insufficient, and further research is warranted. Of note, *sod3* expression decreases with age in healthy adult alveolar type II epithelial cells [176]. Likewise, age-related changes in murine lung *sod3* expression have been reported and exacerbate endotoxin-induced acute lung injury [83,151]. Considering that SARS-CoV-2 hospitalizations and mortality both increase with age [177,178,179], further translational studies regarding pulmonary EC-SOD expression are warranted.

In a conflicting report, plasma EC-SOD *activity* was higher in the serum of patients with ARDS compared to healthy controls [180]. Interestingly, activated neutrophils express high levels of surface-bound cathepsin G [181], and cathepsin G proteolytically destabilizes tetrameric EC-SOD, generating monomers that lack the heparin-binding domain [182], which could increase plasma EC-SOD activity. It is important to note that this study was underpowered [180]. Additionally, CuZn-SOD and Mn-SOD activities were also elevated in ARDS, suggesting that cell lysis may contribute to increased plasma SOD activity [180].

In patients with diabetes, plasma concentrations of EC-SOD were found to be inversely associated with the incidence of myocardial infarction and all-cause mortality [183]. Analogous to findings in diabetic cardiovascular disease, measurements of EC-SOD levels and reactive oxygen species using electron paramagnetic resonance spectrometry in ARDS may reflect endothelial and epithelial injury, processes central to ARDS pathophysiology [111]. Such measurements could potentially be used for disease severity stratification or ARDS phenotyping and to guide precision therapy or serve as biomarkers for treatment response. Measuring *tissue* EC-SOD levels may also have clinical utility, given the association between chronic kidney disease and reduced EC-SOD levels on renal biopsy [134].

ARDS is a biologically heterogeneous syndrome with distinct inflammatory phenotypes, commonly described as hyperinflammatory and hypoinflammatory [184,185]. The hyperinflammatory phenotype is associated with increased endothelial injury and oxidative stress, processes in which EC-SOD plays a critical protective role by limiting superoxide and preserving nitric oxide bioavailability; loss of EC-SOD function in this setting could exacerbate vascular dysfunction and lung injury. In contrast, the hypoinflammatory phenotype may have a lower oxidative burden. Although direct assessments of EC-SOD across ARDS phenotypes are currently lacking, these observations suggest that phenotype-specific evaluation of EC-SOD and oxidative stress pathways may help identify patient subgroups most likely to benefit from targeted antioxidant or EC-SOD-modulating therapies.

## 4. Therapeutics

The mainstay of ARDS treatment is lung-protective ventilation and conservative fluid management. To date, no pharmacologic therapy has been shown to reduce mortality in patients with ARDS [85,186]. Despite promising results in preclinical models, pharmacologic therapies have failed to show benefit when tested in critically ill patients with ARDS. Non-specific antioxidant treatments, including N-acetylcysteine and vitamin C, did not improve outcomes in ARDS [187,188,189].

In ARDS, levels of glutathione in alveolar fluid are reduced, while oxidized glutathione levels are increased [190], suggesting increased oxidative stress and a potential benefit of antioxidant treatment. Direct administration of glutathione is largely ineffective because it is metabolized extracellularly before cellular uptake; however, N-acetylcysteine can effectively increase intracellular glutathione because it is metabolized into cysteine, the rate-limiting precursor for glutathione synthesis. In small clinical trials, patients with COVID-19-associated ARDS received intravenous N-acetylcysteine or placebo. Treatment with N-acetylcysteine did not improve 28-day mortality, ICU length of stay, ventilator-free days, or ARDS severity [188,189].

Vitamin C is a small, water-soluble molecule that acts as a one- or two-electron reducing agent for many radicals and oxidants. In sepsis, plasma concentrations of reduced vitamin C are decreased [191]. Additionally, low vitamin C levels correlate directly with survival and inversely with multiple organ failure [192]. In preclinical investigations using endotoxin-induced models of acute lung injury and sepsis, treatment with vitamin C decreased inflammatory and prothrombotic responses, attenuated lung vascular injury, and improved survival [193]. The CITRIS-ALI trial was a randomized, double-blind, placebo-controlled, multicenter study that examined whether high-dose vitamin C vs. placebo improved outcomes in patients with sepsis and ARDS [187]. There were no significant differences between the vitamin C and placebo groups in modified Sequential Organ Failure Assessment scores, markers of inflammation, all-cause mortality, ventilator-free days, ICU-free days, or hospital-free days [187].

EC-SOD has been extensively studied in preclinical models of lung injury, resulting in a large body of literature supporting its protective effects in endotoxin-, bleomycin-, infection-, hyperoxia-, hypoxia-, and radiation-induced models of lung injury. For instance, preclinical data from a murine hypoxia model demonstrated that pretreatment with aerosolized recombinant human EC-SOD (rhEC-SOD) reduced mortality, lung injury severity, and systemic oxidative stress [194]. Co-administration of zinc has been shown to enhance endocytosis of rhEC-SOD, decreasing NF-κβ and STAT3 activation [195]. To date, there is no data regarding rhEC-SOD in human patients, but recombinant SOD1 has been studied in neonates [196,197,198,199]. Administration of subcutaneous bovine CuZn-SOD in premature neonates with respiratory distress syndrome decreased time on non-invasive mechanical ventilation and the prevalence of bronchopulmonary dysplasia at 12 months [196]. Intratracheal administration of recombinant human CuZn-SOD (rhSOD) has been shown to decrease markers of acute lung injury in premature infants with respiratory distress syndrome but did not decrease the prevalence of bronchopulmonary dysplasia [198]. Of note, patients only received one dose of rhSOD, and illness severity was significantly higher in one experimental group and approached significance in the other compared to placebo [197]. A second study showed a similar reduction in markers of acute lung injury with repeat intratracheal rhSOD dosing but again failed to decrease the prevalence of bronchopulmonary dysplasia [197]. Improvement in clinical outcomes after rhSOD treatment approached significance at one year [199]. General limitations of recombinant SOD include antigenicity, a short circulating half-life, and large molecular size [200]. Of note, intranasal treatment with SOD1-containing polyketal microparticles successfully reduced mortality and markers of acute lung injury in a murine hyperoxia model [201], indicating that microparticles may improve delivery. Additionally, a recent Phase I trial showed that subcutaneous administration of rhSOD had a favorable safety profile and pharmacokinetics in healthy volunteers, supporting its potential as a therapy for diseases driven by excess superoxide [202] (Figure 1).

Synthetic SOD mimetics have been developed to circumvent challenges with native SOD administration. Table 1 lists select SOD mimetics that have been tested in preclinical models of acute lung injury, as described below. The first SOD mimetics included Fe-containing compounds [203] and Mn–desferrioxamine derivatives [204,205]. There are three general classes of Mn-containing SOD mimetics: Mn (III)-metalloporphyrins, Mn (III)-salen complexes, and Mn (II)-pentaazamacrocyclic ligand-based SOD mimetics [200]. Mn-based metalloporphyrins are capable of scavenging superoxide, hydrogen peroxide, peroxynitrite, and lipid peroxyl free radicals [200]. Examples of Mn (III)-metalloporphyrins include MnTE-2-PyP and Mn (III) mesotetrakis (di-*N*-ethylimidazole) porphyrin (AEOL 10150) (Table 1). Pretreatment with subcutaneous MnTE-2-PyP significantly attenuated platelet activation, pulmonary neutrophilia, and accumulation of platelet–neutrophil aggregates in the lungs of mice with *S. aureus* pneumonia [165]. MnTE-2-PyP also protected against weight loss and reduced histologic acute lung injury scores [165], providing further evidence that MnTE-2-PyP is protective against acute lung injury. Of note, intratracheal administration of MnTE-2-PyP did not provide the same protection against *S. aureus*-induced acute lung injury [165]. In a separate study, pretreatment with subcutaneous AEOL 10150 decreased hemorrhage-induced lung lipid peroxidation and NF-κβ activation in mice [150] (Figure 1).

Mn (III)-salen complexes have SOD and catalase activity [200,206,207,208]. Examples of salen SOD mimetics include EUK-8 and EUK-189 (Table 1). In an endotoxemia swine model, pretreatment with EUK-8 was protective against LPS-induced arterial hypoxemia, pulmonary arterial hypertension, and pulmonary edema [208]. Pretreatment with EUK-8 also reduced neutrophil levels in bronchoalveolar lavage fluid and oxidative stress, as evidenced by a reduction in lung malondialdehyde content [208]. A separate study showed that pretreatment of human alveolar epithelial cells with EUK-8 and EUK-189 restored catalase and glutathione peroxidase activities, thereby attenuating ROS production [206]. Additionally, EUK-8 and EUK-189 reduced activation of ROS-dependent signaling pathways involved in NF-κβ and IRF-3 activation [206]. Of note, EUK treatment was protective against lipid peroxidation even after RSV infection was established and significantly reduced viral replication [206].

Mn (II)-pentaazamacrocyclic ligand-based SOD mimetics are also referred to as selective SOD mimetics because they catalytically remove superoxide without reacting with other oxidizing species [200]. In a murine cecal ligation and puncture sepsis model, intraperitoneal administration of the selective SOD mimetic avasopasem manganese (AVA or GC4419) (Table 1) reduced serum cytokine levels (IL-1β, IL-6, TNF-α, and MDA), as well as cytokine expression in lung tissue (IL-1β, IL-6, and TNF-α) [209]. Treatment with AVA also reduced histologic acute lung injury scores and improved natural antioxidant activity [209]. In a separate study, pretreating rat alveolar macrophages with the selective SOD mimetic, M40403 (Table 1), inhibited *Escherichia coli* endotoxin-induced cytokine production, suppressed NF-κβ activation, and decreased O_2_^•−^ production [99].

**Table 1:** **Pharmacologic and pharmacokinetic characteristics of select SOD mimetics**. t_1/2_ denotes elimination half-life in murine models, Cmax denotes the maximum observed concentration in murine models, and n/a indicates information not readily available.

Mimetic Name	SOD Activity (log kcat)	t½ Plasma (hrs)	Plasma C*max* (ug/g)	Clinical Trial(s)
MnTE-2-Pyp	7.79 [210]	76.9 [210]	17.74 [210]	Phase1/2 as topical agent
AEOL 10150	7.83 [211]	6.6 [211]	4.046 [211]	Phase 1
M40403	7.08 [212]	n/a	n/a	Phase 2
EUK-189	5.78 [212]	n/a	n/a	Phase 1 as topical agent
EUK-8	5.78 [212]	n/a	n/a	n/a
GC4419 or AVA	7.3 [213]	n/a	n/a	Phases 1 and 3

Alternatively, mRNA technology and gene therapies have been developed. Aerosolized EC-SOD mRNA complexed with lipofectamine reduced bacterial load, alveolar leukocytosis, and cytokine-induced neutrophil chemoattractant-1 (CINC-1) levels in rats with *E. coli* pneumonia [158]. Similarly, adenovirus-mediated gene transfer significantly increased EC-SOD production in mice, attenuating markers of endotoxin-induced acute lung injury [148] (Figure 1).

It is important to note that therapeutic benefits are often observed only in animals pretreated with EC-SOD therapies or SOD mimetics. Nevertheless, this observation does not entirely rule out therapeutic potential in humans during disease processes that predispose to the development of ARDS. Sepsis, hemorrhage, or trauma often require resuscitation with large volumes of fluid or blood products and lead to inflammation and pulmonary edema. The prophylactic efficacy of SOD mimetics could be assessed in such scenarios. Additionally, the development of novel point-of-care assays may help identify patient subgroups in whom EC-SOD may be useful as a prophylactic agent. It is also important to note that combination therapy may be superior to monotherapy, as the addition of the anti-neutrophil agent Antileukinate was complementary to EC-SOD in reducing markers of acute lung injury in a murine hyperoxia model [149].

## 5. Polymorphisms and Genetic Variants of EC-SOD

Polymorphisms in the *sod3* gene have emerged as key modifiers of antioxidant defense and disease susceptibility, as genetic variation can alter EC-SOD expression, catalytic function, and tissue distribution, thereby affecting extracellular redox homeostasis. There is growing evidence linking these variants to altered oxidative stress responses and the pathogenesis of cardiovascular, inflammatory, and pulmonary diseases.

The naturally occurring single-nucleotide polymorphism (SNP), rs1799895, results in a single amino acid substitution (R213G) within the heparin-binding domain of EC-SOD [214,215,216,217]. This arginine-to-glycine substitution at position 213 disrupts the alpha-helical structure of the heparin-binding domain, significantly reducing EC-SOD affinity for extracellular matrix components, including heparan sulfate and type I collagen, and increasing plasma levels 10–30-fold [214,215,216,217,218,219,220,221,222]. Importantly, the enzymatic activity of EC-SOD remains unchanged in carriers of the R213G polymorphism [214,215,218]. Substitution of Arg^213^ with Gly^213^ also inhibits furin-mediated cleavage of the heparin-binding domain [38] and reduces enzymatic degradation by neutrophil proteases, including trypsin [223].

The prevalence of the R213G variant is relatively low within studied populations; reported ranges are 4–6% in Asian populations [216,217], 2–3% in European populations [215,224], and 1.1% in Xhosa populations [221]. As such, studying the relationship between the R213G variant and ARDS is inherently challenging. For example, one study did not find an association between the R213G variant and time on the ventilator or mortality in patients with ARDS [225]; however, it was underpowered due to the low prevalence of the R213G variant in this population [215,224]. Additional studies with larger sample sizes to address the low prevalence of EC-SOD polymorphisms are warranted. In a more prevalent disease, chronic obstructive pulmonary disease (COPD), the R213G variant has been shown to improve lung function, decrease hospitalizations, and reduce mortality in [226,227]. Similarly, the R213G variant is associated with smaller declines in forced expiratory volume in one second (FEV1) over time, suggesting that the mutation improves lung function in individuals without COPD as well [228]. Importantly, the R213G mutation is also associated with an increased risk of coronary artery disease [222,229,230] and diabetic neuropathy [231].

In the ARDS study referenced above, the authors identified a novel G-C-C-T (4691-5360-5955-5982) *sod3* haplotype block that was associated with significant reductions in time on the ventilator and mortality [225]. The prevalence of an alternative C-T-C-T haplotype within the same block was also significantly higher in patients with ARDS compared to healthy controls; however, it was not associated with reduced ventilator time or mortality [225].

Additional *sod3* SNPs have been identified, including Ala40Thr (rs2536512) [232], E1/I1 (rs8192287/rs8192288) [233], and Phe131Cys (rs2855262) [234]. Of these, Ala40Thr has the greatest clinical relevance. In one study, the mean allele frequency of Ala40Thr was significantly higher in patients with type 2 diabetes compared to healthy controls and correlated with reduced insulin sensitivity, earlier age at diagnosis, and hypertension [235]. A second study demonstrated that the Ala40Thr mutation was associated with reduced FEV1 in pediatric patients with asthma [236]. Ala40Thr has also been identified as an independent risk factor for severe selective fetal growth restriction–complicated pre-eclampsia [237] and hypertension [238]. The E1/I1 mutations are linked and associated with mild reductions in forced vital capacity (FVC) [233]. A similar trend was identified between the E1/I1 mutation and FVC in another study, but it was underpowered and failed to reach significance [228]. Research regarding the Phe131Cys mutation is limited. However, the mean allele frequency was 5.6% in a relatively large Mediterranean population and therefore warrants further research [234]. Collectively, these studies demonstrate the potential impact of various *sod3* variants on disease pathogenesis, including ARDS.

## 6. Conclusions and Future Directions

Accumulating evidence positions EC-SOD as a central regulator of extracellular redox homeostasis in the lung and a critical modifier of the inflammatory response in ARDS. Through its localization to the extracellular matrix and cell surfaces, EC-SOD mitigates superoxide-driven oxidative damage, preserves endothelial and epithelial integrity, limits neutrophil recruitment and activation, and attenuates downstream processes such as glycocalyx degradation, NET formation, and microvascular thrombosis. Experimental models consistently demonstrate that loss of EC-SOD exacerbates lung injury and mortality, whereas restoration or augmentation of EC-SOD activity confers robust protection. Despite compelling preclinical findings, EC-SOD-based strategies have yet to be translated into effective clinical therapies, highlighting the need to better define patient selection, optimal delivery approaches, and therapeutic timing. Future investigations should focus on clarifying the regulation of EC-SOD expression and post-translational modifications during critical illness, resolving conflicting clinical observations regarding circulating EC-SOD levels, and determining how genetic variants influence ARDS susceptibility and outcomes. Additionally, emerging approaches—including SOD mimetics and mRNA-based delivery—offer promising avenues to overcome limitations associated with recombinant protein administration. Together, these efforts may enable targeted modulation of extracellular redox signaling and pave the way for novel, mechanism-driven therapies for ARDS.

## Data Availability

No new data were created or analyzed in this study. Open-access online databases, including the Integrated Human Lung Cell Atlas, the Neonatal Compressed Atlas of the Lung, and COVIDome, were used to generate Figure 2 and Figure 3.
